# Ruxolitinib Alleviates Renal Interstitial Fibrosis in UUO Mice

**DOI:** 10.7150/ijbs.39024

**Published:** 2020-01-01

**Authors:** Yu Bai, Wei Wang, Ping Yin, Jian Gao, Lei Na, Yu Sun, Zhuo Wang, Zhongbo Zhang, Chenghai Zhao

**Affiliations:** 1 Department of Pathophysiology, College of Basic Medical Science, China Medical University, Shenyang, China.; 2 Department of Nephrology, Shengjing Hospital, China Medical University, Shenyang, China.; 3 Center of Laboratory Technology and Experimental Medicine, China Medical University, Shenyang, China.

**Keywords:** Ruxolitinib, Jak, Stat3, mTOR, TGF-β1

## Abstract

Ruxolitinib is a selective inhibitor of Jak1/2. Downstream signaling pathways of Jak, such as Stat3 and Akt/mTOR, are overactivated and contribute to renal interstitial fibrosis. Therefore, we explored the effect of Ruxolitinib on this pathological process. Unilateral ureteral obstruction (UUO) models and TGF-β1-treated fibroblasts and renal tubular epithelial cells were adopted in this study. Ruxolitinib was administered to UUO mice and TGF-β1-treated cells. Kidneys from UUO mice with Ruxolitinib treatment displayed less tubular injuries compared with those without Ruxolitinib treatment. Ruxolitinib treatment suppressed fibroblast activation and extracellular matrix (ECM) production in UUO kidneys and TGF-β1-treated fibroblasts. Ruxolitinib treatment also blocked epithelial-mesenchymal transition (EMT) in UUO kidneys and TGF-β 1-treated renal tubular epithelial cells. Moreover, Ruxolitinib treatment alleviated UUO-induced inflammation, oxidative stress and apoptosis. Mechanistically, Ruxolitinib treatment attenuated activation of both Stat3 and Akt/mTOR/Yap pathways. In conclusion, Ruxolitinib treatment can ameliorate UUO-induced renal interstitial fibrosis, suggesting that Ruxolitinib may be potentially used to treat fibrotic kidney disease.

## Introduction

Renal fibrosis is a hallmark of most forms of progressive renal disease 1. The unilateral ureteral obstruction (UUO) method, involving ligation of the ureter, most commonly of the left one, has been widely adopted to establish animal models with interstitial fibrosis. The kidney of the ligated ureter is conventionally termed as the obstructed kidney, which exhibits several important events such as mechanical stretching, activation of renin-angiotensin-aldosterone system (RAS), loss of renal epithelial cells, inflammation, oxidative stress, macrophage infiltration and fibroblast activation, together leading to the final fibrosis 2. Histologically, the obstructed kidney is characterized by tubular dilation, interstitial expansion, loss of renal parenchyma, infiltration of inflammatory cells, and accumulation of extracellular matrix (ECM) 2.

Some molecular mechanisms have been identified involved in renal interstitial fibrosis. Signaling pathways such as Notch, Wnt and Hedgehog, which are crucial for kidney development, were shown overactivated and to play positive roles in UUO-induced fibrosis 3, 4, 5, 6. Moreover, Erk and Akt signaling pathways were revealed to have a pro-proliferative effect on myofibroblasts by affecting molecules involved in cell cycle such as c-Myc, cyclin D1 and p21 7, 8. Recently, the mammalian target of rapamycin (mTOR) was found implicated in fibroblast proliferation and activation 9, 10, 11, 12. Activation of Stat3 signaling may be another mechanism underlying renal interstitial fibrosis. Expression of phosphorylated Stat3 was enhanced in both tubular epithelial cells and interstitial fibroblasts after UUO 13. Furthermore, blockade of Stat3 pathway was shown to inhibit fibroblast activation in obstructive nephropathy 14, 15.

Ruxolitinib is a potent and selective inhibitor of Jak1/2. It has been approved for the treatment of myelofibrosis, a neoplasm characterized by bone marrow fibrosis, and in which Jak/Stat pathway is over-activated. Ruxolitinib ameliorates disease-related symptoms, improves health-related quality of life, and increases patient survival 16, 17, 18. Notably, a phase 3 study demonstrated that 15.8% patients with Ruxolitinib treatment had improved fibrosis 19.

Based on the above findings, in the present study we explored the effect of Ruxolitinib on UUO-induced renal interstitial fibrosis. We found that Ruxolitinib treatment suppressed fibroblast activation and reduced ECM deposition. Mechanistically, Ruxolitinib treatment attenuated Stat3, Akt, and mTOR signaling pathways in both obstructed kidneys and* in vitro* activated fibroblasts.

## Results

### Ruxolitinib alleviates renal damage

**Figure 1:**
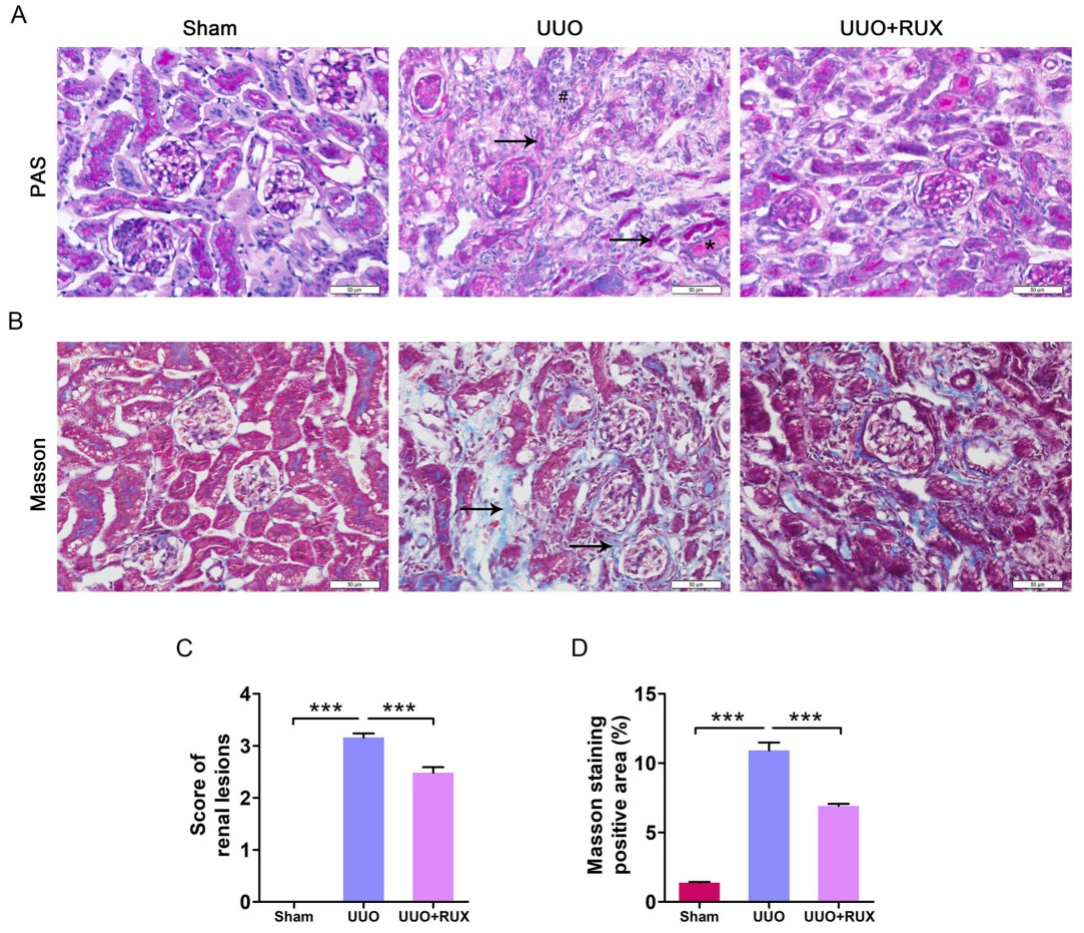
** Ruxolitinib treatment alleviated renal damage in UUO mice. (A)** Histological changes were assessed by PAS staining. ↑: Tubular atrophy; #: Inflammatory cell infiltration; *: Cast formation. **(B)** Fibrosis was assessed by Masson′s trichrome staining. ↑: Fibrosis. **(C)** Renal lesions were scored. (D) The percent of positive area by Masson′s trichrome staining was quantified. Mean ± SEM, n=5. ***p<0.001. RUX: Ruxolitinib. Scalebar, 50 µm.

UUO was used to establish mouse models of obstructive nephropathy. After two weeks, PAS and Masson′s trichrome staining were used to evaluate renal damage and fibrosis. The obstructed kidneys from UUO mice without Ruxolitinib treatment (later called UUO kidneys) exhibited severe structural disorders, characterized by tubular dilation and atrophy, intratubular cast formation, inflammatory cell infiltration, and ECM deposition (Figure 1A-D). However, kidneys from UUO mice with Ruxolitinib treatment displayed remarkably less tubular injuries and ECM deposition, indicating Ruxolitinib treatment alleviated UUO-induced renal damage (Figure 1A-D).

### Ruxolitinib ameliorates ECM deposition

**Figure 2:**
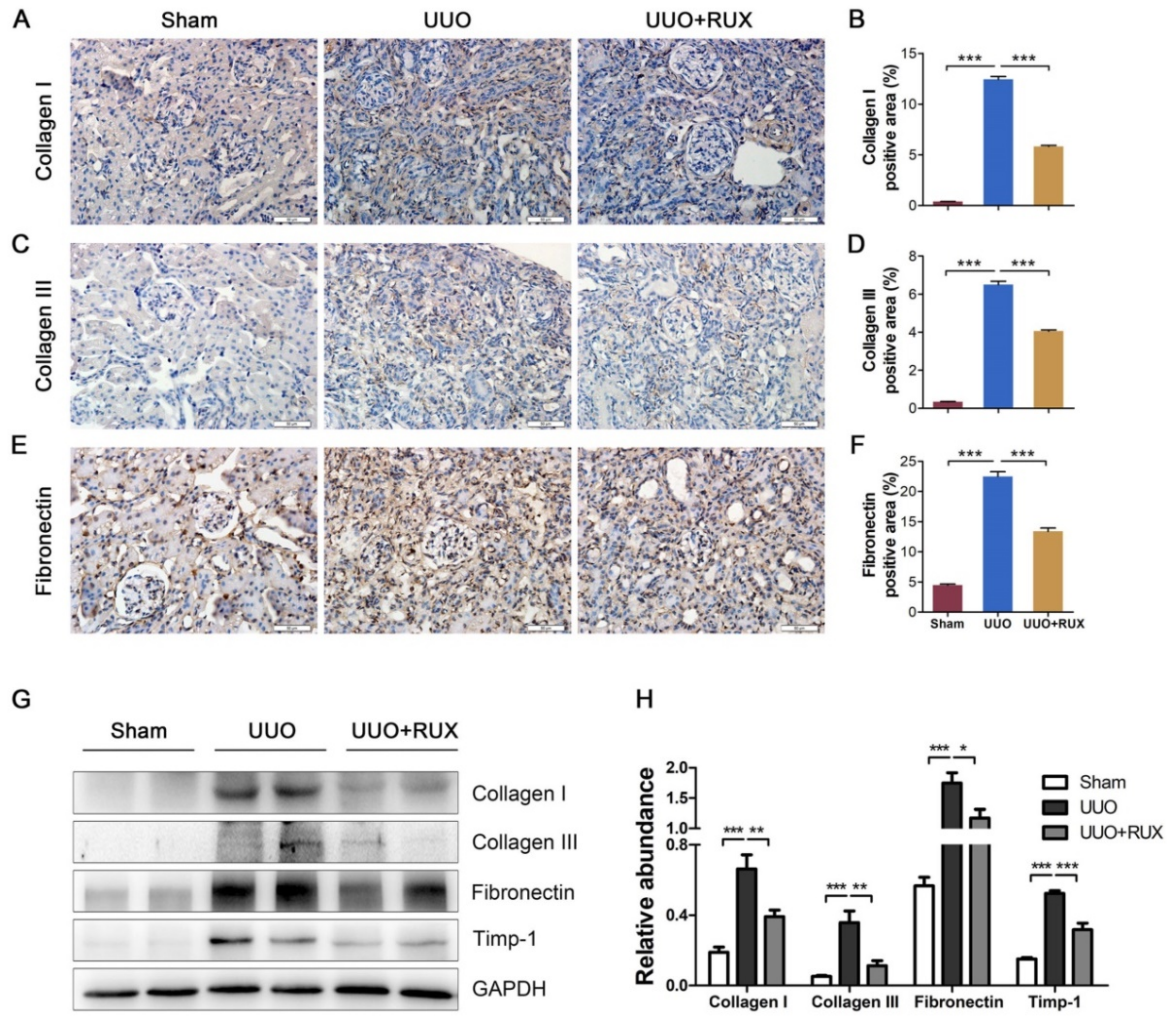
** Ruxolitinib treatment ameliorated ECM deposition in UUO kidneys. (A-F)** Expression of Collagen I, Collagen III and Fibronectin in kidney tissue sections was detected by Immunohistochemistry, and the percent of positive area was quantified. Scale bar, 50μm. **(G-H)** Expression of Collagen I, Collagen III, Fibronectin and Timp-1 in kidney tissue lysates was detected by Western blot, and quantified by densitometry. Mean ± SEM, n=5. *p<0.05, **p<0.01, ***p<0.001. RUX: Ruxolitinib.

To evaluate the effect of Ruxolitinib on UUO-induced fibrosis, we analyzed the deposition of several ECM including Collagen I, Collagen III and Fibronectin. Immunohistochemistry staining showed that UUO kidneys expressed much higher levels of Collagen I (Figure 2A-B), Collagen III (Figure 2C-D) and Fibronectin (Figure 2E-F) compared with normal control (Sham group), and indicating UUO successively induced renal interstitial fibrosis. Ruxolitinib treatment attenuated the deposition of all these ECM (Figure 2A-F). Western blot detection similarly revealed Ruxolitinib treatment reduced Collagen I, Collagen III and Fibronectin expression in UUO kidneys (Figure 2G-H). Furthermore, Western blot detection indicated that Ruxolitinib treatment suppressed UUO-induced metallopeptidase inhibitor 1 (Timp-1) upregulation (Figure 2G-H), suggesting that Ruxolitinib treatment may not only reduce ECM production, but also promote ECM degradation.

### Ruxolitinib blocks renal fibroblast activation

**Figure 3:**
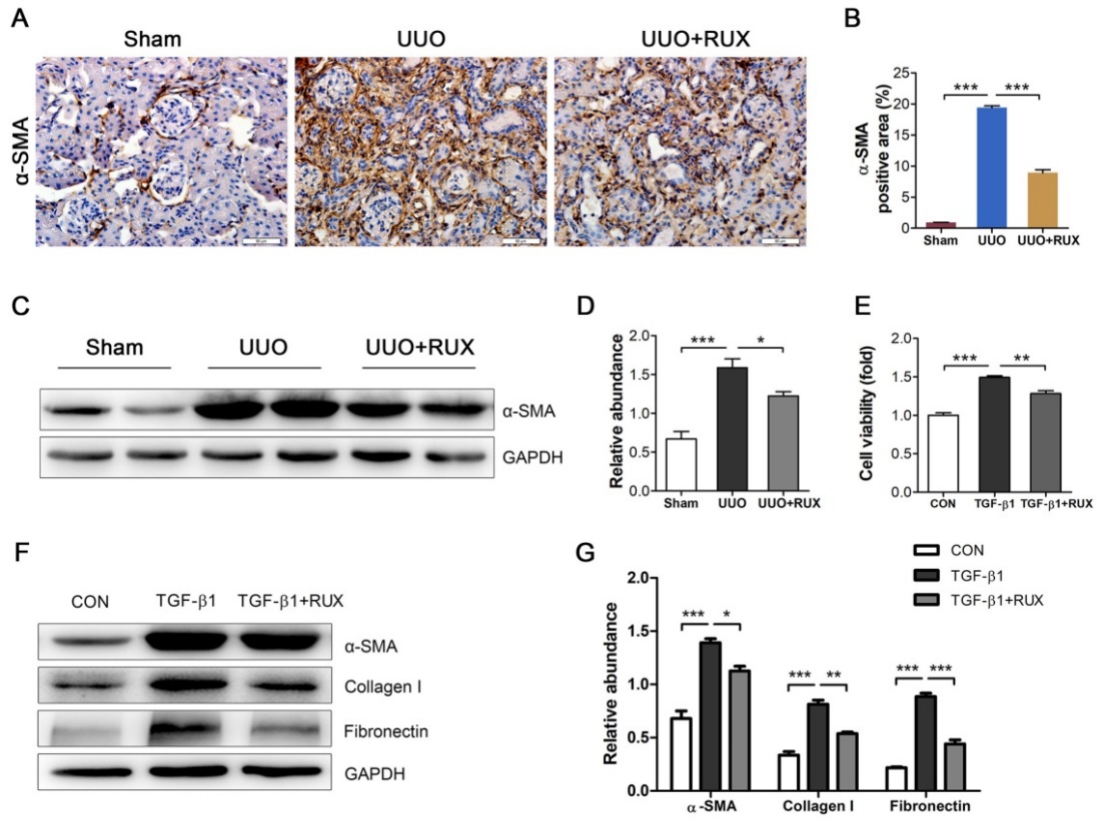
** Ruxolitinib treatment blocked renal fibroblast activation in UUO kidneys and TGF-β1 -induced NRK-49F cells. (A-B)** Expression of α-SMA in kidney tissue sections was detected by Immunohistochemistry, and the percent of positive area was quantified. Mean ± SEM, n=5. Scale bar, 50μm. **(C-D)** Expression of α-SMA in kidney tissue lysates was detected by Western blot, and quantified by densitometry. Mean ± SEM, n=5. **(E)** Proliferation of NRK-49F cells was assessed by MTT methods. Mean ± SEM from three independent experiments. **(F-G)** Expression of α-SMA, Collagen I and Fibronectin in NRK-49F cell lysates was detected by Western blot, and quantified by densitometry. Mean ± SEM from three independent experiments. *p<0.05, **p<0.01, ***p<0.001. RUX: Ruxolitinib; CON: Control.

Fibroblast activation is crucial for renal interstitial fibrosis. Activated fibroblasts or myofibroblasts are sources of ECM. We assessed the effect of Ruxolitinib on fibroblast activation using UUO models and TGF-β1 -treated NRK-49F cells. Staining of α-SMA by Immunohistochemistry revealed much more activated fibroblasts in UUO kidneys compared with normal control. Ruxolitinib treatment notably blocked UUO-induced fibroblast activation, which was indicated as a decrease in α-SMA expression (Figure 3A-B). This finding was further confirmed by Western blot detection (Figure 3C-D). TGF-β1 was used to activate NRK-49F cells. MTT measurement showed that Ruxolitinib treatment suppressed TGF-β1 -induced NRK-49F cell proliferation (Figure 3E). Furthermore, Ruxolitinib treatment downregulated TGF-β1 -induced α-SMA expression (Figure 3F-G). Consistently, Ruxolitinib treatment counteracted TGF-β1 -induced Collagen I and Fibronectin expression in NRK-49F cells (Figure 3F-G).

### Ruxolitinib suppresses renal tubular cell EMT

**Figure 4:**
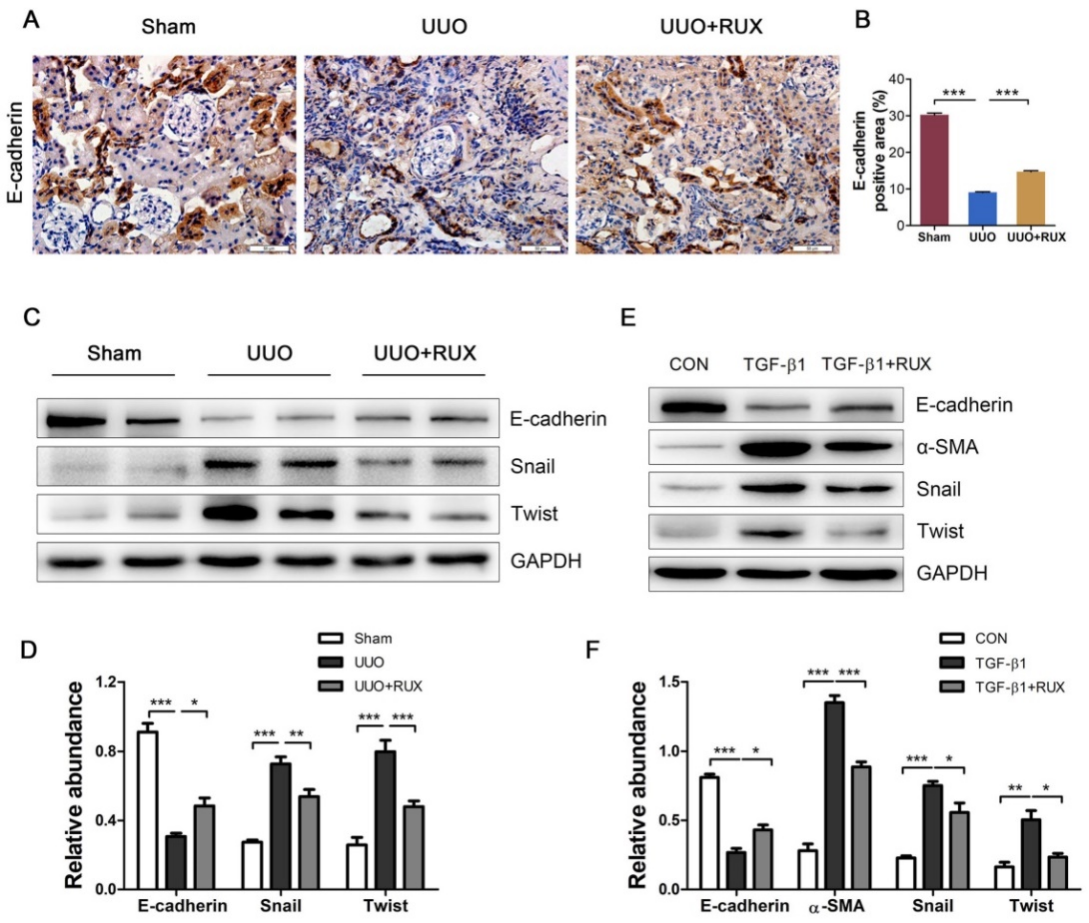
** Ruxolitinib treatment suppressed renal tubular cell EMT in UUO kidneys and TGF-β1 -induced NRK-52E cells. (A-B)** Expression of E-cadherin in kidney tissue sections was detected by Immunohistochemistry, and the percent of positive area was quantified. Mean ± SEM, n=5. Scale bar, 50μm. **(C-D)** Expression of E-cadherin, Snail and twist in kidney tissue lysates was detected by Western blot, and quantified by densitometry. Mean ± SEM, n=5. **(E-F)** Expression of E-cadherin, α-SMA, Snail and Twist in NRK-52E cell lysates was detected by Western blot, and quantified by densitometry. Mean ± SEM from three independent experiments. *p<0.05, **p<0.01, ***p<0.001. RUX: Ruxolitinib; CON: Control.

Epithelial-mesenchymal transition (EMT) endows tubular epithelial cells a mesenchymal phenotype, with increased migratory capacity and ECM production 2. We next investigated the effect of Ruxolitinib on renal tubular cell EMT using UUO models and TGF-β1 -treated NRK-52E cells. Both Immunohistochemistry and Western blot detection showed that E-cadherin, an epithelial marker, was remarkable downregulated in UUO kidneys, while Ruxolitinib treatment effectively restored its expression (Figure 4A-D). Western blot detection also revealed that Ruxolitinib treatment inhibited UUO-induced upregulation of Snail and Twist, two EMT transcription factors (Figure 4C-D). TGF-β1 was used to induce NRK-52E cell EMT. Ruxolitinib treatment compensated for TGF-β1 -induced loss of E-cadherin, and blocked TGF-β1 -induced upregulation of α-SMA, Snail and Twist (Figure 4E-F).

### Ruxolitinib inhibits inflammatory responses

**Figure 5:**
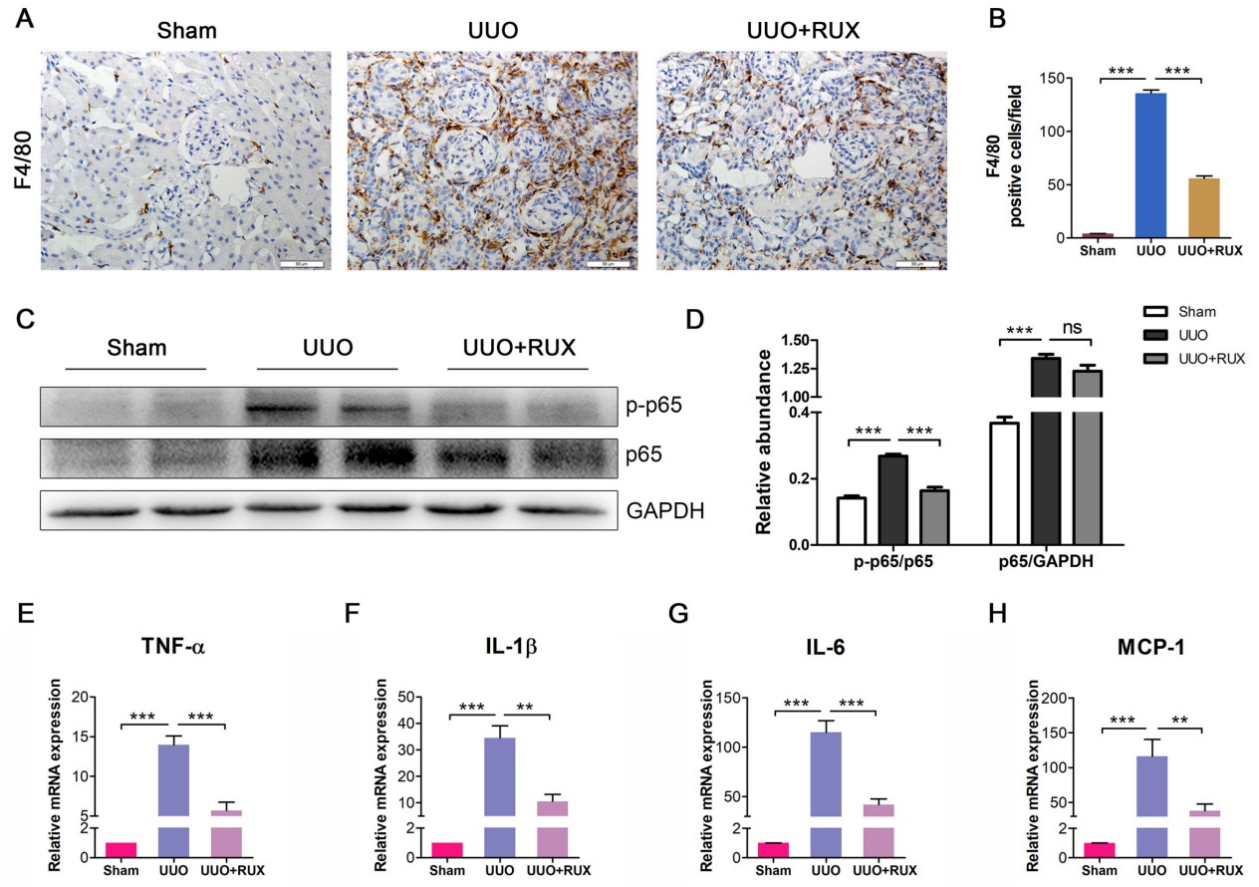
** Ruxolitinib treatment inhibited UUO kidney inflammation. (A-B)** Expression of F4/80 in kidney tissue sections was detected by Immunohistochemistry, and the positive cells were counted. Scale bar, 50μm. **(C-D)** Expression of p-p65 and p65 in kidney tissue lysates was detected by Western blot, and quantified by densitometry. **(E-H)** Levels of TNF-α, IL-1β, IL-6 and MCP-1 mRNA in kidney tissue lysates were detected by Real-time PCR, and quantified by 2^-ΔΔCT^ method. Mean ± SEM, n=5. **p<0.01, ***p<0.001. ns: no significance. RUX: Ruxolitinib.

Inflammation is a hallmark of obstructed kidney characterized by inflammatory cell infiltration and inflammatory cytokine production. We examined F4/80 expression to assess macrophage infiltration. As shown by Immunohistochemistry, F4/80 expression level is higher in UUO kidneys compared with normal control. Ruxolitinib treatment reduced F4/80 expression in UUO kidneys significantly, indicating macrophage infiltration was effectively inhibited (Figure 5A-B). NFκB signaling was activated in UUO kidneys, whereas Ruxolitinib treatment suppressed p-65 phosphorylation (Figure 5C-D). We further evaluated the effect of Ruxolitinib on the expression of several inflammatory cytokines. Real-time PCR detection revealed that Ruxolitinib treatment reduced levels of TNF-α, IL-1β, IL-6 and MCP-1/CCL2 mRNA (Figure 5E-H).

### Ruxolitinib reduces renal tubular epithelial cell apoptosis

**Figure 6:**
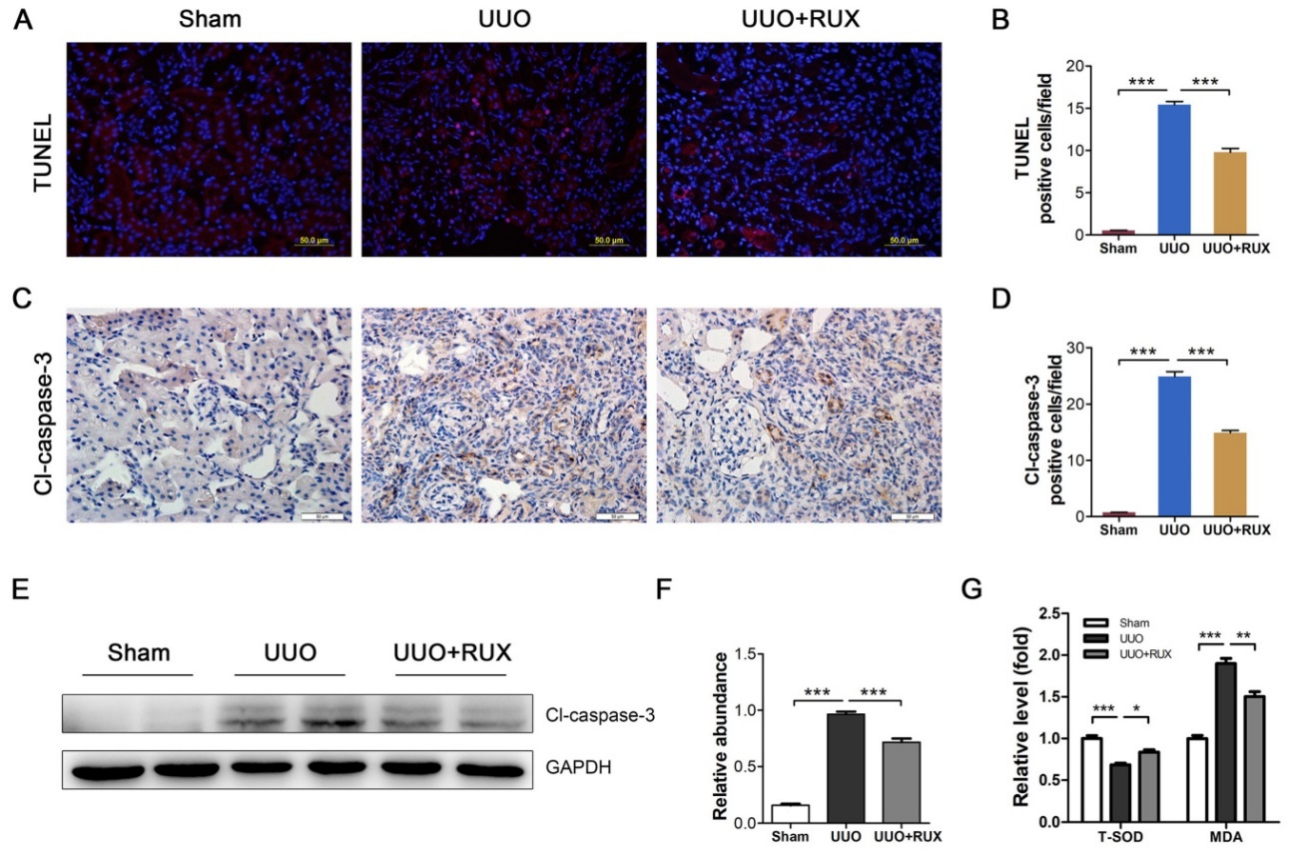
** Ruxolitinib treatment reduced renal tubular cell apoptosis and oxidative stress in UUO kidneys. (A-B)** Apoptotic cells in kidney tissue sections were detected by TUNEL staining, and counted. Scalebar, 50 µm. **(C-D)** Expression of cleaved caspase-3 in kidney tissue sections was detected by Immunohistochemistry, and the positive cells were counted. Scalebar, 50 µm. **(E-F)** Expression of cleaved caspase-3 in kidney tissue lysates was detected by Western blot, and quantified by densitometry. **(G)** Relative levels of T-SOD and MDA in kidney tissues were detected by corresponding kits. Mean ± SEM, n=5. *p<0.05, ***p<0.001. RUX: Ruxolitinib; Cl-caspase-3: Cleaved caspase-3.

Renal tubular epithelial cell apoptosis is an important event in obstructed kidney. TUNEL staining was used to determine cell apoptosis. It was shown that apoptotic cells increased in UUO kidneys. Ruxolitinib treatment ameliorated the situation (Figure 6A-B). Cleaved caspase-3 was subsequently examined. As shown by both Immunohistochemistry and Western blot, cleaved caspase-3 expression increased in UUO kidneys, while decreased after Ruxolitinib treatment (Figure 6C-F). As oxidative stress is one mechanism underlying cell apoptosis, we thereafter investigated levels of Malondialdehyde (MDA) and total Superoxide dismutase (T-SOD). As expected, UUO resulted in an increase in MDA, and a reduction in T-SOD; Ruxolitinib treatment restored T-SOD and MDA towards normal level (Figure 6G-H).

### Ruxolitinib attenuates Akt/mTOR/Yap pathway

**Figure 7:**
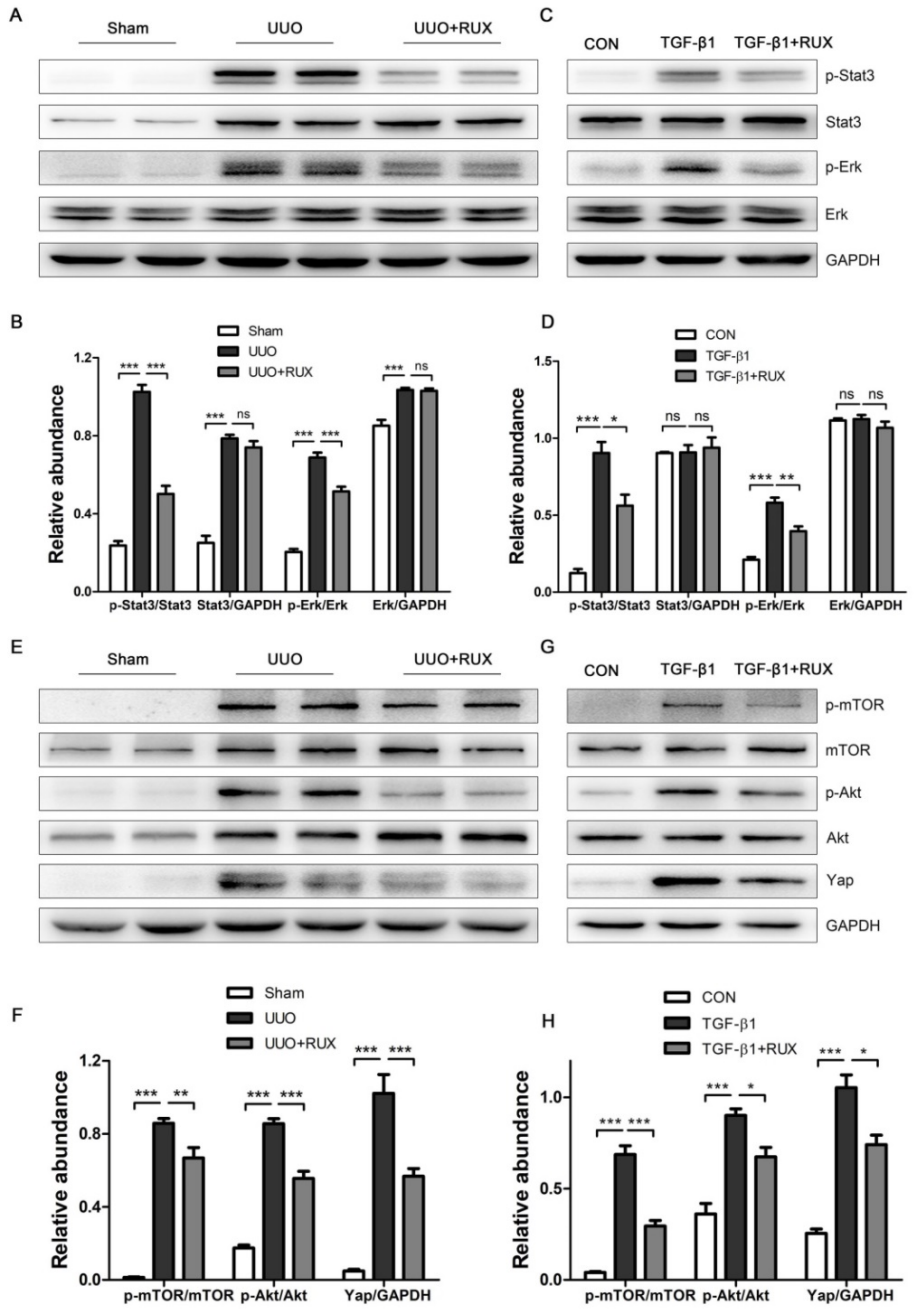
** Ruxolitinib treatment attenuated Stat3 and Akt/mTOR/Yap pathways in UUO kidneys and TGF-β1 -induced NRK-49F cells. (A-B)** Expression of p-Stat3, Stat3, p-Erk and Erk in kidney tissue lysates was detected by Western blot, and quantified by densitometry. Mean ± SEM, n=5. **(C-D)** Expression of p-Stat3, Stat3, p-Erk and Erk in NRK-49F cell lysates was detected by Western blot, and quantified by densitometry. Mean ± SEM from three independent experiments. **(E-F)** Expression of p-mTOR, mTOR, p-Akt, Akt and Yap in kidney tissue lysates was detected by Western blot, and quantified by densitometry. Mean ± SEM, n=5. **(G-H)** Expression of p-mTOR, mTOR, p-Akt, Akt and Yap in NRK-49F cell lysates was detected by Western blot, and quantified by densitometry. Mean ± SEM from three independent experiments. *p<0.05, **p<0.01, ***p<0.001, ns: no significance. RUX: Ruxolitinib; CON: Control.

We finally explored signaling pathways targeted by Ruxolitinib. UUO kidneys exhibited increased expression of phosphorylated Stat3 and Erk. Ruxolitinib treatment significantly suppressed Stat3 and Erk phosphorylation (Figure 7A-B). Similarly, Ruxolitinib treatment inhibited Stat3 and Erk phosphorylation in TGF-β1 -treated NRK-49F cells (Figure 7C-D). We further analyzed downstream molecules of Jak signaling such as Akt and mTOR which have been shown involved in obstructed kidney 9, 10. Consistent with these reports, UUO kidneys overexpressed Akt, p-Akt, mTOR and p-mTOR. Ruxolitinib treatment reduced Akt and mTOR phosphorylation (Figure 7E-F). Recently, yes-associated protein (Yap) was shown as a downstream of mTOR and involved in UUO kidney 12, 20. Indeed, Ruxolitinib treatment inhibited Yap expression (Figure 7E-F). Moreover, Ruxolitinib treatment suppressed Akt and mTOR phosphorylation and Yap expression in TGF-β1 -treated NRK-49F cells (Figure 7G-H).

## Discussion

Activated fibroblasts expressing α-SMA are conventionally called myofibroblasts 1. Myofibroblasts produce ECM; therefore they are crucial for organ fibrosis. Study by LeBleu and colleagues demonstrated that in UUO kidneys, 50% of myofibroblasts originate from local resident fibroblasts through proliferation, and 35%, 10% and 5% of myofibroblasts arise from bone marrow differentiation, endothelial-to-mesenchymal transition and EMT, respectively 21. Our study revealed that Ruxolitinib treatment reduced α-SMA expression in both UUO kidneys and TGF-β1 -treated NRK-49F cells, and consistently, Ruxolitinib treatment resulted in a reduced ECM production; Moreover, Ruxolitinib treatment interfered with TGF-β1 -induced NRK-49F cell proliferation. Together, these findings indicate that Ruxolitinib has a potentiality to suppress fibroblast activation or myofibroblast generation.

As renal tubular epithelial cell EMT contributes to UUO-induced renal interstitial fibrosis, we observed the effect of Ruxolitinib on E-cadherin expression. Just as expected, UUO resulted in a decreased E-cadherin expression, which was partially recovered by Ruxolitinib treatment. Furthermore, Ruxolitinib treatment blocked TGF-β1 -induced E-cadherin downregulation in NRK-52E cells. Transcription factors snail and twist have been shown involved in renal tubular epithelial cell EMT 22, 23. Our study demonstrated that Ruxolitinib treatment suppressed snail and twist upregulation in both UUO kidneys and TGF-β1 -treated NRK-52E cells. However, study by Pang and colleagues indicated that a specific Stat3 inhibitor, S3I-201, has no effect on UUO-induced snail overexpression 15. These findings suggest that Ruxolitinib inhibits snail upregulation independent on Stat3.

UUO kidneys overproduce chemokines and their receptors 24, 25, 26. These chemokines are responsible for the recruitment of inflammatory cells, which, when activated, produce more cytokines to sustain and enhance inflammation. MCP-1 recruits macrophages into renal interstitium 27, 28, 29. Our study showed that Ruxolitinib treatment reduced MCP-1 expression as well as macrophage infiltration in the obstructed kidneys. In contrast, blockade of Stat3 by S3I-201 failed to suppress UUO-induced MCP-1 upregulation, but still reduced macrophage infiltration, suggesting that other cytokines were involved in macrophage recruitment 15. Besides growth factor such as TGF-β1, cytokine such as IL-6 also contributes to fibroblast activation and fibrosis 30. A recent study showed that blockade of IL-6 improved UUO-induced fibrosis 31.

Several factors have been identified to induce tubular epithelial cell apoptosis, including mechanical stretch, Angiotensin II, TGF-β1, Fas/FasL and oxidative stress 32, 33. In our study, Ruxolitinib treatment reduced expression of TNF-α, which was shown to stimulate apoptosis in UUO kidneys 34, 35. Moreover, Ruxolitinib treatment attenuated the oxidative stress response. It has been shown that UUO impairs renal antioxidant enzyme activation 36. Ruxolitinib treatment recovered T-SOD level in the obstructed kidneys, and consistently, alleviated UUO-induced apoptosis.

Our study finally identified that Ruxolitinib treatment attenuated Akt/mTOR pathway. Both mTOR complex 1 (mTORC1) and mTORC2 were activated in UUO kidneys 9, 10. Furthermore, Yap was revealed to mediate mTORC2-induced renal interstitial fibrosis 12. Increasing evidence indicated that Yap functioned as a pro-fibrotic factor in kidneys, and targeting Yap improved renal interstitial fibrosis 20, 37, 38, 39. Ruxolitinib treatment inhibited Yap expression in UUO kidneys, further suggesting that Akt/mTOR/Yap is a potential target signaling by Ruxolitinib.

In summary, Ruxolitinib treatment alleviated inflammation and oxidative stress in UUO kidneys, and suppressed fibroblast activation, tubular cell EMT and ECM production in both UUO kidneys and *in vitro* cultured cells. Mechanistically, Ruxolitinib treatment blocked UUO or TGF-β1 -induced activation of both Stat3 and Akt/mTOR/Yap pathways. These findings indicate that Ruxolitinib treatment can ameliorate UUO-induced renal interstitial fibrosis, and suggest that Ruxolitinib could be potentially used to treat fibrotic kidney disease.

## Materials and Methods

### Chemicals and antibodies

Ruxolitinib phosphate (Jakavi, Novartis) and Ruxolitinib (INCB018424; Selleck chemicals) were used for *in vivo* and *in vitro* experiment, respectively. Antibodies to collagen I (ab34719), collagen III (ab7778), Fibronectin (ab2413), Timp-1 (ab86482), and α-SMA (ab124964) were purchased from Abcam. Antibodies to E-cadherin (#3195), Snail (#3879), Twist (#46702), F4/80 (#70076), mTOR (#2972), p-mTOR (#2971), Akt (#9272), p-Akt (Ser473, #9271), Stat3 (#12640), p-Stat3 (Tyr705, #9145), Erk 1/2 (#4695), p-Erk 1/2 (#4370), and Yap (#14074) were purchased from Cell Signaling Technology. Antibodies to p-NFκB p65(sc-33020) and NFκB p65(sc-109) was purchased from Santa Cruz Biotechnology. TUNEL assay kit (KGA7061) for apoptosis was purchased from KeyGEN BioTECH (Nanjing, China).

### UUO models and Ruxolitinib treatment

Male C57BL/6 mice (Beijing Huafukang Biotechnology, China) that weighed 22-24g were randomly assigned to three groups with 5 mice in each group as follows: (1) Sham-operated mice with vehicle (Sham); (2) UUO mice with vehicle (UUO); (3) UUO mice treated with Ruxolitinib (UUO+RUX). To establish UUO model, mice were given general anesthesia by intraperitoneal injection of pentobarbital (50mg/kg body weight). The left ureter was exposed via a left flank incision, ligated with 4-0 silk at two points, and cut between the 2 ligation points. The Sham-operated group had no ligation. For *in vivo* experiments, Ruxolitinib was dissolved in PEG300/dextrose 5% in a ratio of 1:3 (PEG/dex) and administered to mice by oral gavage at a dosage of 30 mg/kg twice daily for 14 days immediately after UUO or Sham-operation. The Sham and UUO group received PEG/dex alone as vehicle. The mice were sacrificed, and the left kidneys were collected at days 14 after surgery. All procedures were performed in accordance with guidelines approved by the Institutional Animal Care and Use Committee of China Medical University.

### PAS and Masson′s trichrome staining

The paraffin-embedded sections were stained with PAS (Solarbio, China, G1281) and Masson's trichrome (Solarbio, China, G1340) to evaluate histological change and fibrosis. Ten non-repeating fields were randomly selected. Tubular lesions were scored from 0 to 4 31. 0: normal; 1: mild (<25% of the cortex); 2: moderate (25~50%); 3: severe (50~75%); 4: extensive damage (>75%). The positive area of Masson's trichrome staining (blue) was calculated with the Image-Pro Plus.

### Cell culture and treatment

Rat fibroblast NRK-49F and rat renal tubular epithelial cell NRK-52E were cultured in Dulbecco's modified Eagle's medium (DMEM) containing 10% fetal bovine serum (FBS) and 1% penicillin/streptomycin at 37°C with 5% CO_2_. 2ng/ml TGF-β1 was used to activate NRK-49F cells or induce NRK-52E cell EMT. These cells were starved for 12 h and then exposed to TGF-β1 with or without 5μM Ruxolitinib for 24 hours.

### Immunohistochemistry

Tissue sections were deparaffinized, hydrated, and incubated with 3% H_2_O_2_ to remove endogenous peroxidase. Then the sections were incubated with primary antibody overnight at 4 ℃, and next with biotinylated secondary antibody for 30 min at 37 °C. Subsequently the sections were stained with DAB, re-stained in hematoxylin, dehydrated, and sealed with cover slides. Ten non-repeating images of each sample were acquired using microscope image system. Positive signals and numbers of positive cells were quantified using Image-Pro Plus software.

### Western blot

Equal amount of protein was isolated by SDS-PAGE, and transferred to PVDF membranes which was blocked by 5% bovine serum albumin or skimmed milk for 2 h at room temperature, and incubated with primary antibody overnight at 4°C. The membrane was subsequently incubated with peroxidase-conjugated goat secondary antibody (1:5000, ZSGB Bio) for 2 h at room temperature. Protein content was determined by SuperSignal™ West Pico PLUS Chemiluminescent Substrate (Thermo Scientific).

### Real-time PCR

Total RNAs were extracted using RNAiso Plus (Takara, Cat#9108), and reversely transcribed into cDNA using PrimeScript™ RT reagent Kit with gDNA Eraser (TaKaRa, Cat#RR047a) according to the instructions. Real-time PCR was carried out using TB Green™ *Premix Ex Taq*™ II (Tli RNaseH Plus) (RR820A, TaKaRa). The primers for TNF-α are, forward: 5′-gcgacgtggaactggcagaag-3′ and reverse: 5′-gccacaagcaggaatgagaagagg-3′; for IL-6, forward: 5′-acttccatccagttgccttcttgg-3′ and reverse: 5′-ttaagcctccgacttgtgaagag-3′; for IL-1β, forward: 5′-tcgcagcagcacatcaacaagag-3′ and reverse: 5′-tgctcatgtcctcatcctggaagg-3′. for MCP-1, forward: 5′-ccactcacctgctgctactcattc-3′ and reverse: 5′-ctgctgctggtgatcctcttgtag-3′. GAPDH was used as internal control. Expression difference was assessed using 2^-ΔΔCT^ method.

### TUNEL

The apoptotic cells in kidney tissue sections were detected using a TUNEL apoptosis detection kit (KeyGEN BioTECH, China, KGA7061) according to the instructions provided by the manufacture. Briefly, paraffin-embedded sections were treated with fresh diluted proteinase K for 20 min at 37 ℃ after deparaffinage and rehydration. TdT enzyme reaction mixture was applied to each slide at 37 ℃ for 60 min. Then, Streptavidin-TRITC was added into the slides at 37 ℃ for 30 min. At last, the slides were reacted with DAPI solution for 10 min at room temperature. The number of TUNEL-positive nuclei per field was counted in 10 non-repeating micrographs for each sample.

### MTT

Cell viability was measured at 48 h using an MTT assay kit (Beyotime, China, C0009). 2,000 Cells in 96-well plate were washed and incubated with MTT staining solution for 4 h. Then Formazan solving solution was added. The cell viability was indicated by the absolution value at 570nm.

### T-SOD and MDA measurement

T-SOD (A001-1) and MDA (A003-1) kits were purchased from Jiancheng Bioengineering Institute (Nanjing, China). Briefly, kidney tissues were prepared into 10% homogenates which were centrifuged at 3,000 rpm for 10 min. The supernatants were collected to measure levels of T-SOD and MDA according to the instructions.

### Statistical analysis

All the experiments were conducted at least three times. GraphPad Prism 7.0 was used to analyze the data, and all data were expressed as the Mean ± SEM. Inter-group comparisons were performed using One-way analysis of variance (ANOVA). Multiple means were compared by Tukey′s test. *P*<0.05 is considered as significant.

## Abbreviations

UUO: unilateral ureteral obstruction
ECM: extracellular matrix
EMT: epithelial-mesenchymal transition
RAS: renin-angiotensin-aldosterone system
mTOR: mammalian target of rapamycin
Timp-1: metallopeptidase inhibitor 1
MDA: malondialdehyde
T-SOD: total superoxide dismutase
Yap: yes-associated protein
mTORC1: mTOR complex 1
